# Supplementary material for: Metabolic Modifications in Terpenoid and Steroid Pathways Triggered by Methyl Jasmonate in Taxus × media Hairy Roots
Source: Plants (Basel). 2022 Apr 20;11(9):1120. doi: 10.3390/plants11091120 (PMC9100385; doi:10.3390/plants11091120)
Supplement: Supplementary file 1 [file plants-11-01120-s001.zip › plants-1653997-supplementary.pdf]

Metabolic Modifications in Terpenoid and Steroid Pathways Triggered by Methyl Jasmonate in *Taxus* x *media* Hairy Roots

Table S1. GC-MS data (retention times and characteristic ions of mass spectra) of identified steroids and triterpenoids.

Compound (peak number)	Formula	Molecular weight	Retention time [min]	Mass spectrum *m/z* (relative intensity)
cholesterol (1)	$C_{27}H_{46}O$	386.6	31.06	386 (26), 107 (50), 105 (48), 91 (57), 81 (54), 79 (46), 69 (47), 57 (87), 55 (73), 43 (100), 41 (55)
campesterol (2)	$C_{28}H_{48}O$	400.6	33.74	400 (30), 107 (51), 105 (55), 95 (49), 83 (45), 81 (64), 71 (62), 57 (77), 55 (77), 43 (100), 41 (52)
stigmasterol (3)	$C_{29}H_{48}O$	412.6	34.52	412 (36), 145 (64), 107 (52), 95 (100), 83 (66), 81 (90), 78 (60), 69 (67), 67 (85), 55 (69)
sitosterol (4)	$C_{29}H_{50}O$	414.7	36.42	414 (29), 145 (54), 107 (59), 105 (60), 95 (54), 91 (49), 81 (57), 57 (68), 55 (70), 43 (100)
isofucosterol (5)	$C_{29}H_{48}O$	412.3	36.78	412 (5), 314 (100), 105 (47), 95 (50), 91 (42), 83 (40), 81 (51), 69 (61), 55 (96), 43 (49)
β-amyrin	$C_{30}H_{50}O$	426.7	37.35	426 (27), 219 (18), 218 (100), 203 (49), 189 (17), 135 (11), 109 (13), 105 (12), 95 (15), 81 (18), 69 (14)
5-campestenenone (6)	$C_{28}H_{46}O$	398.7	37.59	398 (27), 314 (26), 229 (49), 135 (20), 124 (100), 107 (21), 95 (29), 91 (22), 55 (38)
α-amyrin	$C_{30}H_{50}O$	426.7	38.60	426 (4), 219 (18), 218 (100), 203 (20), 189 (19), 135 (17), 133 (15), 122 (16), 119 (15), 95 (16)
tremulone (7)	$C_{29}H_{46}O$	410.7	39.3	410 (32), 187 (27), 174 (100), 161 (37), 159 (26), 91 (28), 57 (28), 55 (37), 43 (44), 41 (28)
4-stigmasten-3-one (8)	$C_{29}H_{48}O$	412.7	40.89	412 (37), 229 (34), 218 (31), 124 (100), 109 (31), 95 (41), 81 (27), 69 (32), 55 (37), 43 (44)
oleanolic acid methyl ester	$C_{31}H_{50}O_3$	470.1	46.37	470 (1), 262 (48), 207 (13), 204 (16), 203 (100), 202 (21), 189 (22), 133 (17), 119 (13), 105 (14
stigmastane-3,6-dione (9)	$C_{29}H_{48}O_2$	428.6	48.02	428 (25), 135 (61), 107 (74), 98 (63), 95 (67), 79 (62), 69 (86), 57 (67), 55 (100), 43 (77), 41 (71)
ursolic acid methyl ester	$C_{31}H_{50}O_3$	470.1	48.96	470 (1), 263 (20), 262 (100), 207 (32), 203 (93), 189 (29), 133 (76), 119 (34), 105 (21), 95 (18)

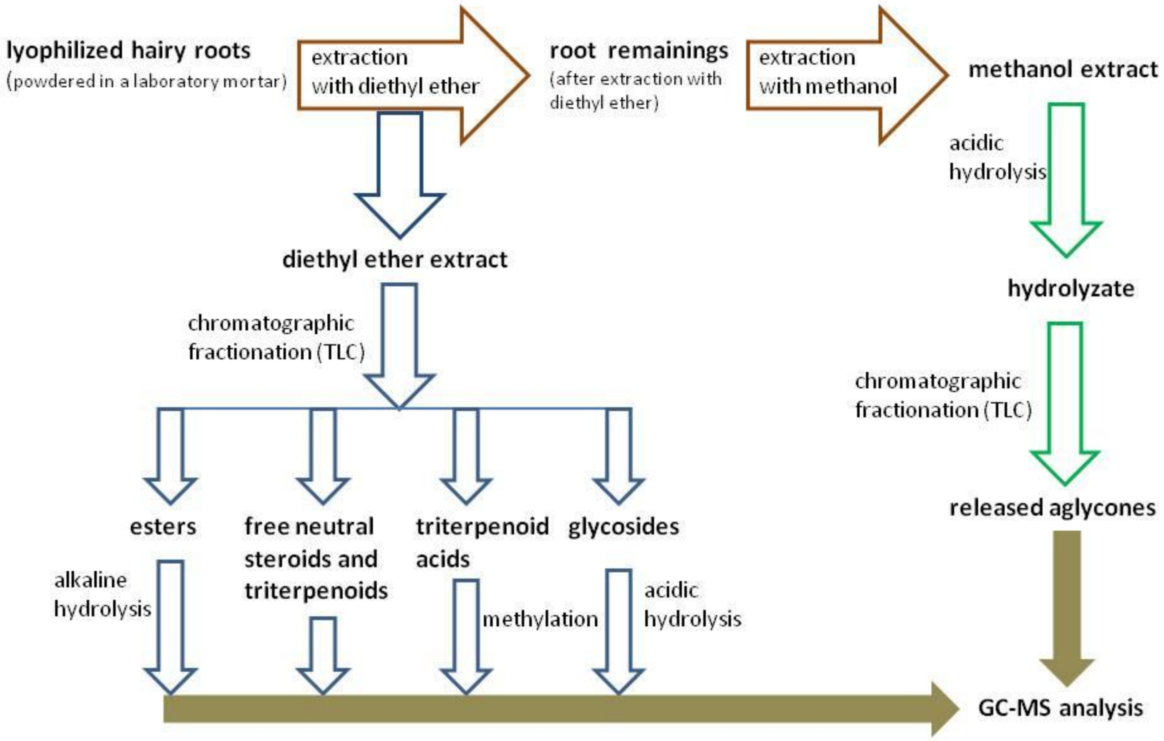

Figure S1. The general scheme of the experimental procedure related to the extraction, separation and analysis of steroids and triterpenoids in *T*. x *media* hairy roots.
